# Supramolecular chemistry II

**DOI:** 10.3762/bjoc.7.181

**Published:** 2011-11-22

**Authors:** Christoph A Schalley

**Affiliations:** 1 Institut für Chemie und Biochemie - Organische Chemie, Freie Universität Berlin, Takustr. 3, 14195 Berlin, Germany

Supramolecular chemistry is a rapidly growing field, which has had remarkable impact on the life sciences on one hand and on materials sciences on the other. In the life sciences, the networks of noncovalent interactions between the constituents of cells, for example, have shifted into the current focus. Self-assembly, templation, self-sorting and multivalent binding all contribute to setting up the extremely complex architecture of a cell. But the same concepts are useful for generating materials with function, when for example the building blocks are programmed appropriately to find their places in a larger, noncovalent architecture. The basis for all these concepts is molecular recognition. Recently, many studies have been devoted to quantifying host–guest interactions, aiming at a more profound understanding of the subtle entropic and enthalpic effects that govern the interactions between host and guest.

A first Thematic Series devoted to supramolecular chemistry was assembled about two years ago and published by the Beilstein Journal of Organic Chemistry [1]. This first series of articles had quite a broad scope ranging from encapsulation and carbohydrate, peptide, anion and ammonium ion binding, through chiral recognition, the formation of pseudorotaxanes and template effects, all the way to allosteric binding to synthetic receptors, crystallographic studies of halogen bonding and the use of polymers for protein binding.

The second series again has a broad scope, as you will discover in the coming months as the series develops. With the second Thematic Series on supramolecular chemistry, we wish to contribute to the endeavor to investigate noncovalently bound complexes and aggregates of every possible kind, thus highlighting the importance of the above-mentioned concepts.

With the now well-known Thematic Series, the Beilstein Journal of Organic Chemistry provides an excellent platform for this aim, in particular since it is a true open access journal.

I would like to thank warmly all authors who have accepted the invitation to contribute to this series and sincerely hope that the readers will enjoy reading the articles that are published within this Thematic Series.

Christoph A. Schalley

Berlin, October 2011
